# Renalase Protects against Renal Fibrosis by Inhibiting the Activation of the ERK Signaling Pathways

**DOI:** 10.3390/ijms18050855

**Published:** 2017-04-27

**Authors:** Yiru Wu, Liyan Wang, Dai Deng, Qidong Zhang, Wenhu Liu

**Affiliations:** Department of Nephrology, Affiliated Beijing Friendship Hospital, Faculty of Kidney Diseases, Capital Medical University, No. 95 Yong An Road, Xi Cheng District, Beijing 100050, China; wyryyyy@163.com (Y.W.); wangliyan7731@sina.com (L.W.); dazziling@163.com (D.D.); yyzqd2007@126.com (Q.Z.)

**Keywords:** renalase, chronic kidney disease, renal interstitial fibrosis, ERK signaling pathway, epithelial–mesenchymal transition

## Abstract

Renal interstitial fibrosis is a common pathway for the progression of chronic kidney disease (CKD) to end-stage renal disease. Renalase, acting as a signaling molecule, has been reported to have cardiovascular and renal protective effects. However, its role in renal fibrosis remains unknown. In this study, we evaluated the therapeutic efficacy of renalase in rats with complete unilateral ureteral obstruction (UUO) and examined the inhibitory effects of renalase on transforming growth factor-β1 (TGF-β1)-induced epithelial–mesenchymal transition (EMT) in human proximal renal tubular epithelial (HK-2) cells. We found that in the UUO model, the expression of renalase was markedly downregulated and adenoviral-mediated expression of renalase significantly attenuated renal interstitial fibrosis, as evidenced by the maintenance of E-cadherin expression and suppressed expression of α-smooth muscle actin (α-SMA), fibronectin and collagen-I. In vitro, renalase inhibited TGF-β1-mediated upregulation of α-SMA and downregulation of E-cadherin. Increased levels of Phospho-extracellular regulated protein kinases (p-ERK1/2) in TGF-β1-stimulated cells were reversed by renalase cotreatment. When ERK1 was overexpressed, the inhibition of TGF-β1-induced EMT and fibrosis mediated by renalase was attenuated. Our study provides the first evidence that renalase can ameliorate renal interstitial fibrosis by suppression of tubular EMT through inhibition of the ERK pathway. These results suggest that renalase has potential renoprotective effects in renal interstitial fibrosis and may be an effective agent for slowing CKD progression.

## 1. Introduction

It is generally recognized that the incidence of chronic kidney disease (CKD) is increasing worldwide and is a public health problem posing a serious threat to human health. Renal interstitial fibrosis (RIF), characterized by excessive deposition of extracellular matrix, is recognized as a common pathological feature of CKD which leads to the development of end-stage renal disease (ESRD), accompanied by progression of renal dysfunction [1]. Accumulating evidence suggests that epithelial–mesenchymal transition (EMT) of tubular epithelial cells contributes significantly to the onset and pathogenesis of RIF [2]. EMT, a process whereby epithelial cells lose their epithelial phenotype and gain attributes of mesenchymal cells, has been implicated in the generation of myofibroblasts and fibroblasts in kidney disease [3]. Of the many factors that trigger EMT, transforming growth factor-β1 (TGF-β1) is considered to be the most potent inducer of EMT in various types of epithelial cells via both Smad-dependent and -independent mechanisms [4]. Therefore, targeting TGF-β1-mediated EMT may ameliorate RIF and the progressive loss of renal function.

Renalase is a recently discovered flavoprotein with oxidoreductase activity, which is expressed in a variety of tissues including the kidney, heart and nervous system [5,6]. In addition to its enzymatic function, renalase may also act as a signaling molecule which can lighten acute kidney injury (AKI) by interacting with cell surface receptors, such as the plasma membrane calcium ATPase isoform PMCA4b [7,8]. The expression level of renalase is significantly diminished in sub-totally nephrectomized rats and patients with ESRD and CKD [9,10,11]. Clinical studies show that renalase deficiency and single-nucleotide polymorphisms in renalase are associated with essential hypertension, coronary heart disease, stroke and diabetes [10,12,13]. Administration of recombinant renalase protects against AKI, contrast-induced nephropathy and cardiac ischemia/reperfusion injury [14,15,16,17]. However, there is no research on whether renalase can reverse EMT and reduce renal interstitial fibrosis.

In the present study, we examined the anti-fibrosis effects of renalase in vivo in a rat model of tubulointerstitial fibrosis induced by unilateral ureteral obstruction (UUO) and in vitro in EMT of human proximal tubular epithelial cells induced by TGF-β1. In addition, we explored whether the effects of renalase effects are mediated by interfering with canonical or non-canonical TGF-β1 signaling pathways through investigation of signaling molecules in vitro.

## 2. Results

### 2.1. Establishment of the UUO Model and Expression of Renalase in Rat Kidneys

**Figure 1:**
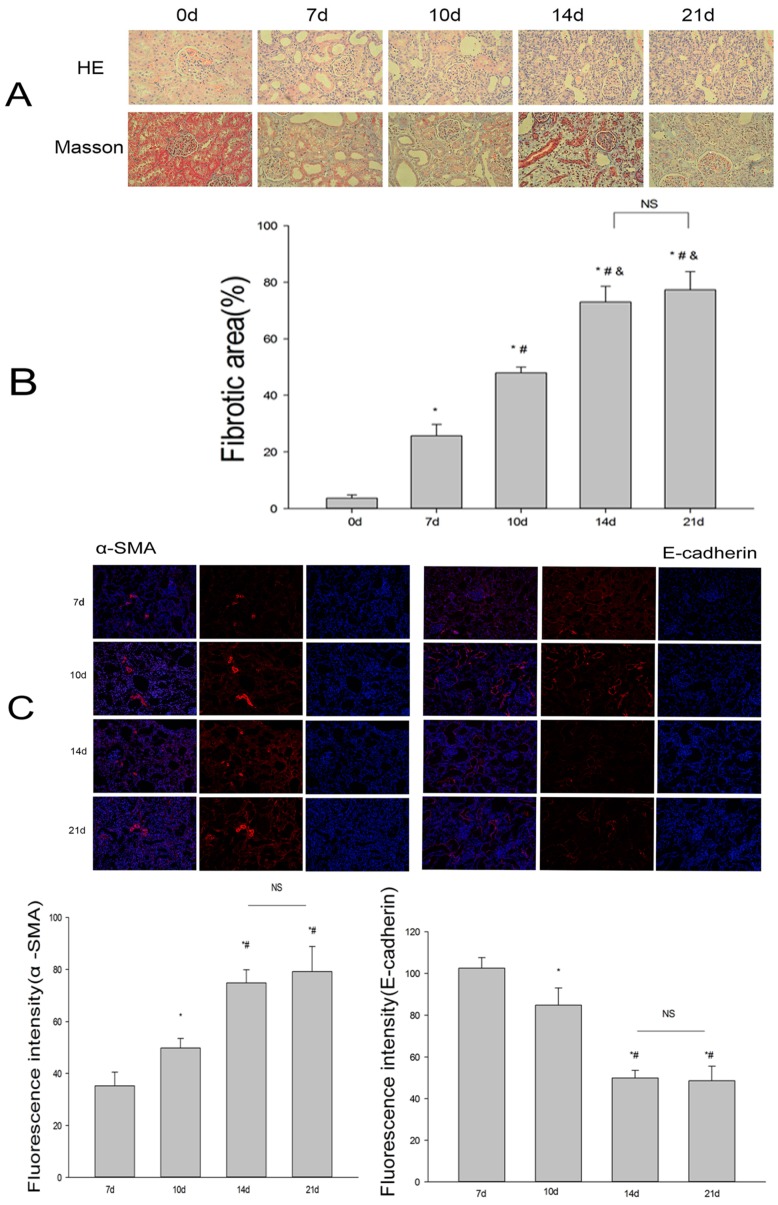

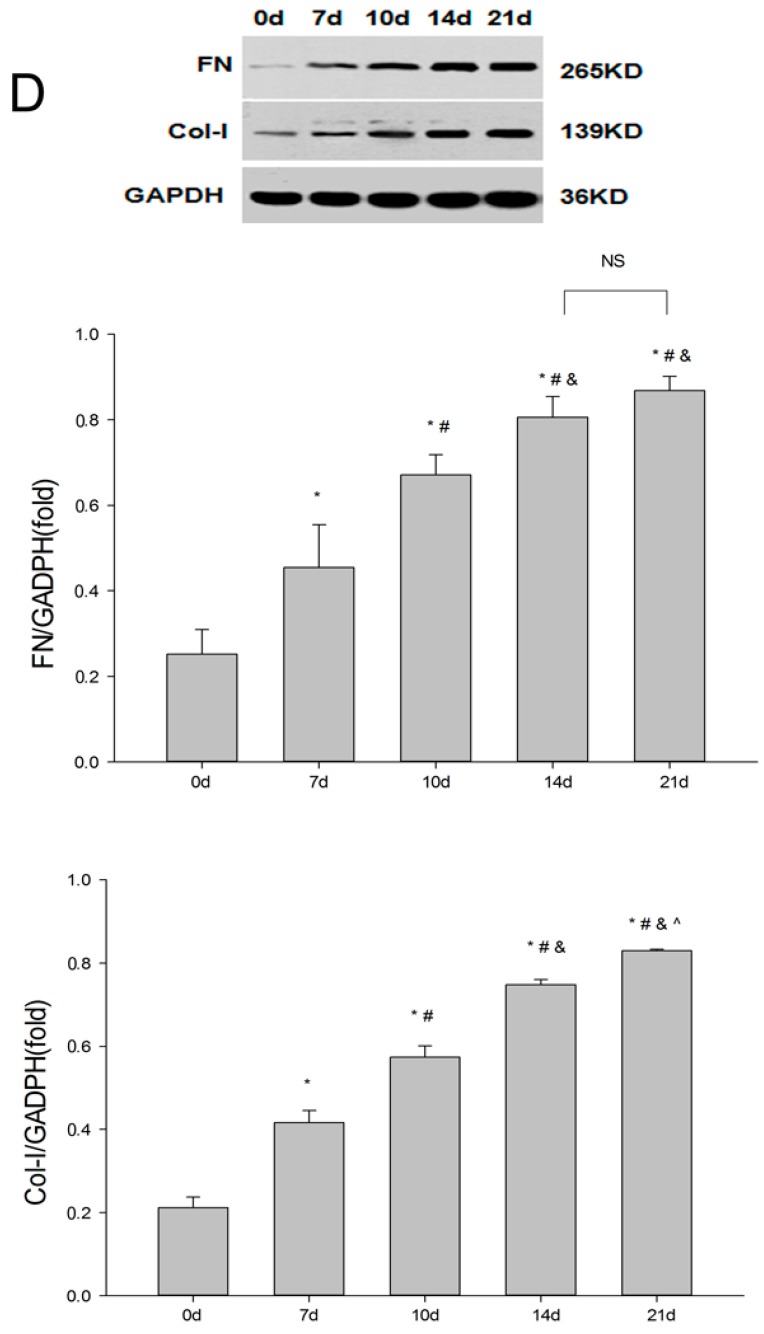

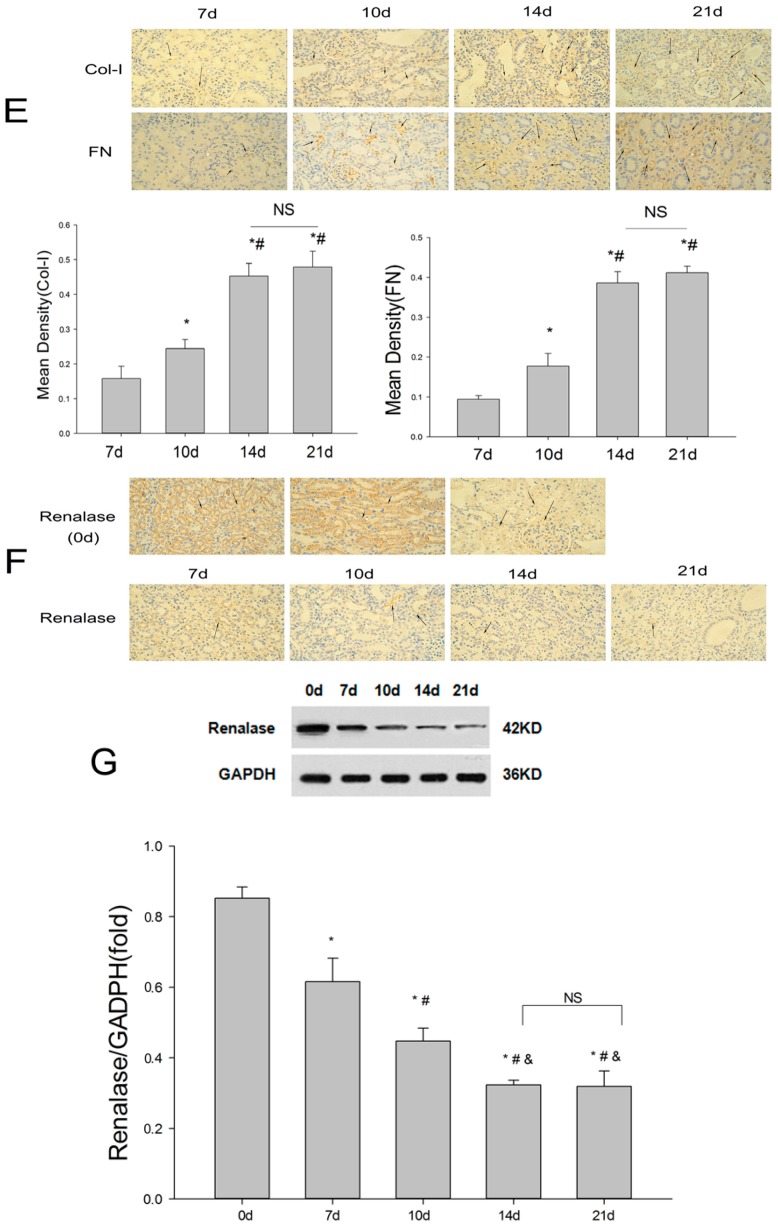

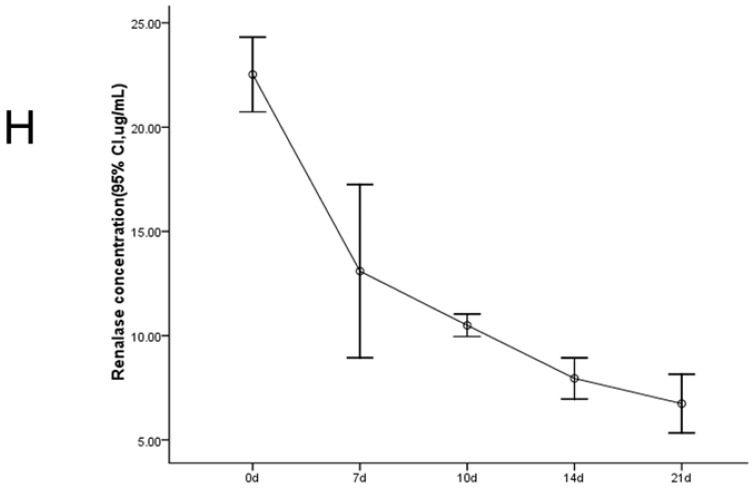
Establishment of the unilateral ureteral obstruction (UUO) model and expression of renalase in rat kidneys. (**A**) Histological changes in kidneys in unilateral ureter ligation. Kidney sections from various groups were subjected to Hematoxylin eosin (H&E) and Masson’s trichrome staining (MTS) at different times after UUO. With the prolongation of obstruction, renal tubular epithelial cell atrophy, luminal expansion, interstitial inflammatory cell infiltration and tubule widening were aggravated and interstitial fibrosis increased gradually; magnification: 40×; (**B**) Quantitative analysis of renal fibrotic lesions in various groups. Renal fibrotic lesions (defined as the percentage of the MTS-positive fibrotic area) were quantified by computer-aided morphometric analyses; (**C**) Immunofluorescence showing the expression of E-cadherin and smooth muscle actin (α-SMA). Representative photomicrographs are shown. With the extension of obstruction, fluorescence intensity of E-cadherin became weak and fluorescence intensity of α-SMA strengthen gradually. The difference was not statistically significant at 14 and 21 days; magnification: 40×; (**D**,**E**) Western blotting and immunohistochemistry demonstrated that, after UUO, the expression of fibronectin and collagen-I increased in a time-dependent manner. The difference was not statistically significant at 14 days and 21 days. The brown/yellow color indicates antibody binding; Arrows refer to positive results in Figure 1E. Magnification: 40×; (**F**) Representative photomicrographs of immunohistochemistry showing the expression and localization of renalase in the ligated kidney. Renalase was mainly distributed in the renal tubules under normal conditions; After UUO, with the aggravation of fibrosis, the expression of renalase was gradually decreased. The difference was not statistically significant in 14 days and 21 days; Arrows refer to positive results in Figure 1E. Magnification: 40×; (**G**) Western blotting and densitometric analysis of renalase protein levels in various groups. The results were consistent with immunohistochemistry; (**H**) The concentration of renalase in plasma. With the prolongation of obstruction, the concentration of renalase in plasma decreased gradually. Results are presented as percentages of control values and are the means ± Standard deviation (SD) of five animals per group. * *p* < 0.05, compared with the control group; ^#^ *p* < 0.05, compared with the 7-day group; ^&^ *p* < 0.05, compared with the 10-day group; ^ *p* < 0.05, compared with the 14-day group; NS: no statistical difference; FN: fibronectin; Col-I: collagen I; GADPH: glyceraldehyde-3-phosphate dehydrogenase; d = days.

At 7, 10, 14, or 21 days after UUO, rats were killed and their kidneys harvested to analyze fibrosis. In the sham group, the structure of the kidney was normal; the renal tubules were arranged closely and orderly, the basement membrane was smooth and continuous, and there was no inflammatory cell infiltration of the interstitial area. After UUO, the kidney on the obstructed side was enlarged while renal parenchyma was thinned. Seven days after the operation, there was a large number of patchy inflammatory cell infiltrates in the renal interstitium, moderate to severe dilatation of the renal tubules, portions of distal tubular atrophy, luminal occlusion, renal tubule epithelial cell swelling and degeneration, and renal interstitial edema and broadening, showing mild fibrous tissue proliferation. Moreover, with prolonged obstruction, the above phenomena were further aggravated (Figure 1A). Masson’s trichrome staining (MTS) revealed that, in the sham group, the linear distribution of blue interstitial collagen fibers can only be seen in the renal tubular basement membrane. In the UUO group, as time passed, the blue interstitial collagen fibers gradually increased, suggesting progressive fibrosis (Figure 1A,B). Moreover, immunofluorescence demonstrated that the expression of E-cadherin decreased while α-SMA increased (Figure 1C). Immunohistochemistry revealed that the expression of fibronectin (FN) and collagen I (Col-I) increased with the time of obstruction (Figure 1E), consistent with western blot data (Figure 1D). However, there was no statistically significant difference in FN between the 14-day group and 21-day group (*p* = 0.242). Given that at 14 days post-obstruction renal fibrosis was already evident, we selected 14 days as the endpoint at which to analyze fibrosis in the subsequent experiments.

To observe the relationship between fibrosis and renalase, we first investigated the expression level of renalase in the kidney. Immunohistochemistry showed that, in 8-week-old rats, renalase was mainly distributed in the renal tubules under normal conditions. There was also significant expression in glomerular mesangial areas (Figure 1F). After UUO, with the aggravation of fibrosis, the expression of renalase was gradually decreased, both in the kidney (Figure 1F,G) and in plasma (Figure 1H). 

### 2.2. Renalase Ameliorates Renal Interstitial Fibrosis in the UUO Model

To investigate the relationship between renalase and RIF in the UUO model, we used an adenovirus to increase the expression of renalase as described in the Materials and Methods. At 3 days after surgery, western blot and immunohistochemistry showed the expression of renalase was significantly decreased in the UUO group compared with the sham group, while in contrast, the expression of renalase was similar in the UUO + Ad-renalase group compared with the sham group (Figure S1A,B). Notably, there was no difference in blood pressure between the groups (data not shown). The expression of renalase in the UUO + Ad-renalase group declined at 7 days after surgery (Figure S1A,B). Thus, adenovirus treatment only restored renalase expression in the kidney for 3 days after surgery (Figure S1A,B). The concentration of renalase in the circulation in the UUO + Ad-renalase group is shown in Figure S1C. The reason that the duration of the action of adenovirus is short may be that the adenovirus was cleared by the body or that the adenovirus itself has become attenuated. Therefore, we reinjected adenovirus once every 3 days in further experiments.

**Figure 2:**
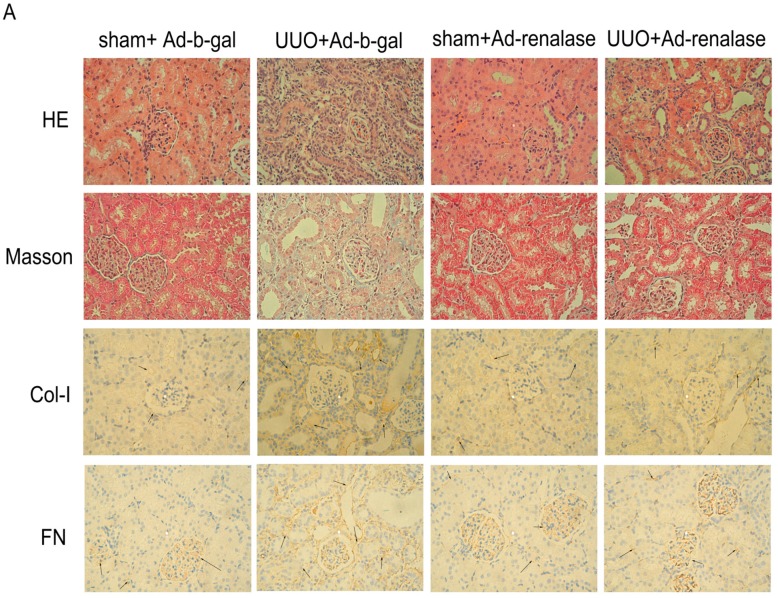

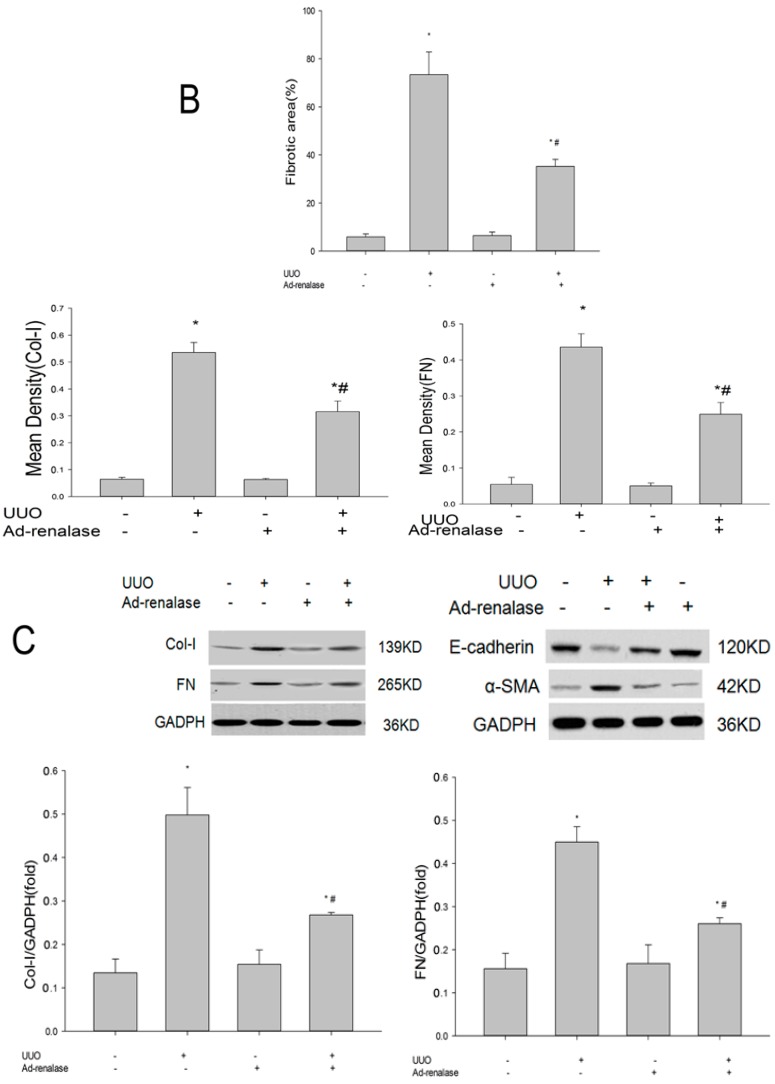

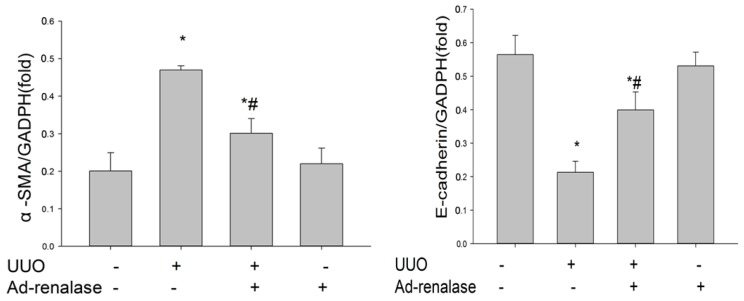
Renalase ameliorates renal interstitial fibrosis in the UUO model. (**A**) Kidney sections from various groups at 14 days after UUO were subjected to H&E, Masson’s trichrome staining (MTS) and immunohistochemistry. Representative micrographs showing that renalase ameliorated renal fibrotic lesions after obstructive injury. Immunohistochemistry showed that renalase reduced fibronectin and collagen I deposition in the obstructed kidney. Arrows refer to positive results. magnification: 40×; (**B**) Quantitative determination of renal fibrotic lesions and quantitative analysis of immunohistochemistry in various groups. Renal fibrotic lesions (defined as the percentage of the MTS-positive fibrotic area) were quantified by computer-aided morphometric analyses. That is, the fibrosis and the expression of FN and Col-I was lightened in UUO + Ad-renalase group compared to UUO + Ad-b-gal group, but was still severe compared with sham groups; (**C**) Western blot showed that renalase inhibited renal expression of α-SMA, fibronectin, and collagen I and increased renal expression of E-cadherin in the ligated kidney at 14 days after UUO. Results are presented as percentages of control values and are the means ± SD of five animals per group. * *p* < 0.05, compared with the sham + Ad-b-gal group; ^#^ *p* < 0.05, compared with the UUO + Ad-renalase group.

In order to observe the degree of interstitial fibrosis, kidney tissues of rats were stained with MTS. Renal interstitial fibrosis in Ad-renalase-treated rats was significantly reduced compared with that in Ad-b-gal-treated UUO rats and UUO-alone rats, as shown in Figure 2A,B. Consistent with the histological data, protein levels of fibrosis markers, including Col-I and FN, were significantly higher in the remnant kidneys of Ad-b-gal-treated UUO rats than in those of Ad-renalase-treated UUO and sham rats (Figure 2A,C). Moreover, EMT was significantly more obvious in the remnant kidneys of Ad-b-gal-treated UUO rats than in those of Ad-renalase-treated UUO and sham rats. Specifically, the expression of α-SMA was higher and the expression of E-cadherin was lower in the remnant kidneys of Ad-b-gal-treated UUO rats than in those of Ad-renalase-treated UUO and sham rats (Figure 2C). However, protein levels of fibrosis markers in Ad-renalase-treated UUO rats were still higher than sham rats (Figure 2A,C).

### 2.3. Renalase Inhibits TGF-β1-Mediated Tubular EMT and Fibrosis In Vitro

**Figure 3:**
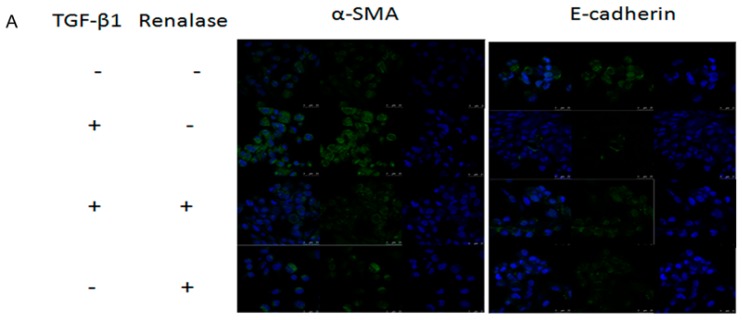

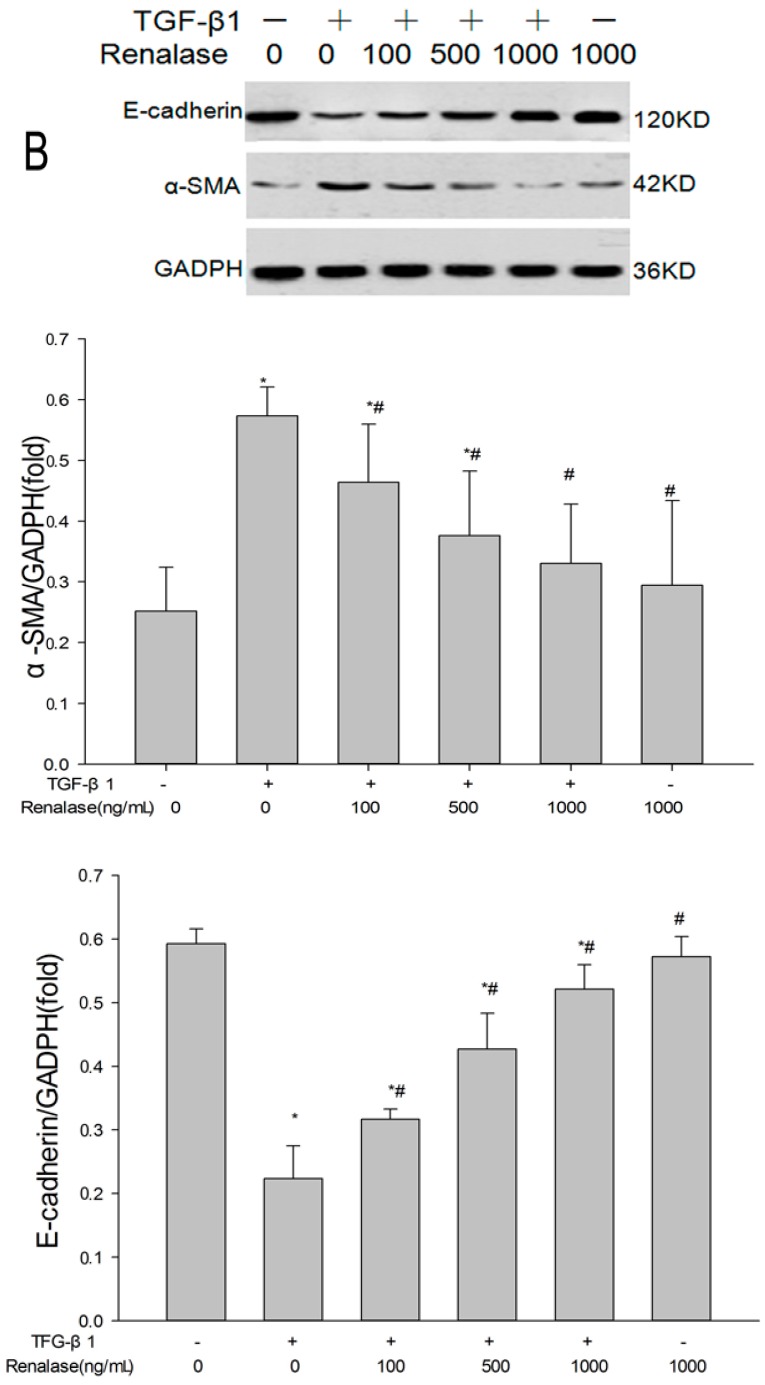

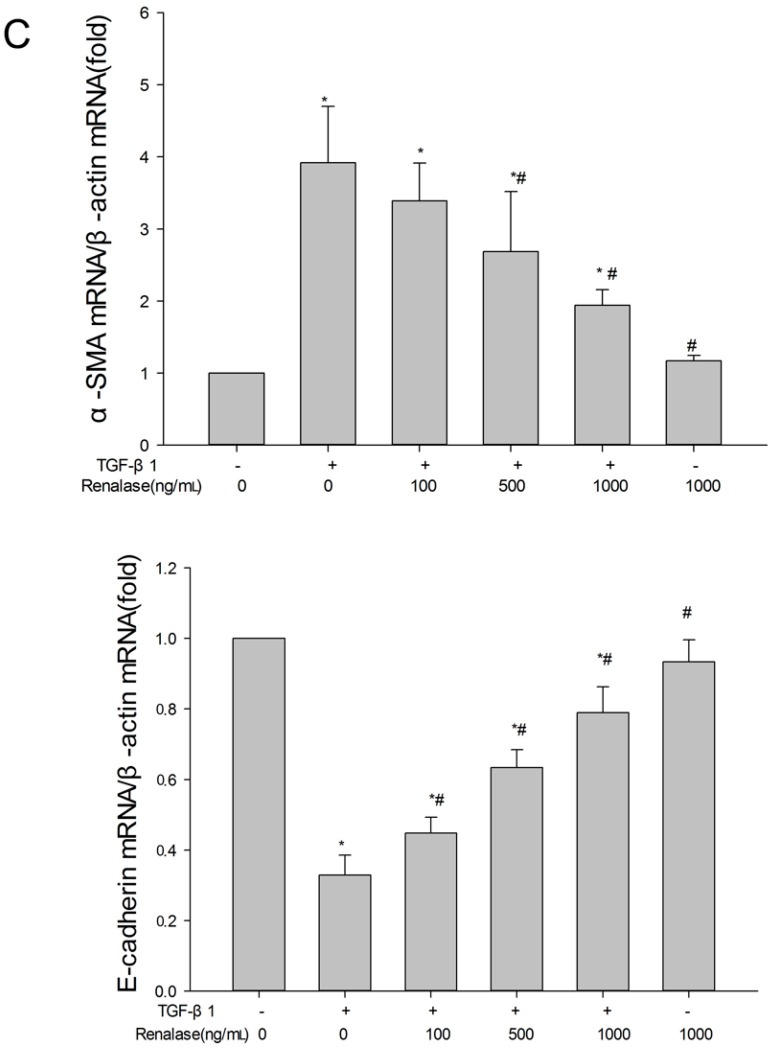

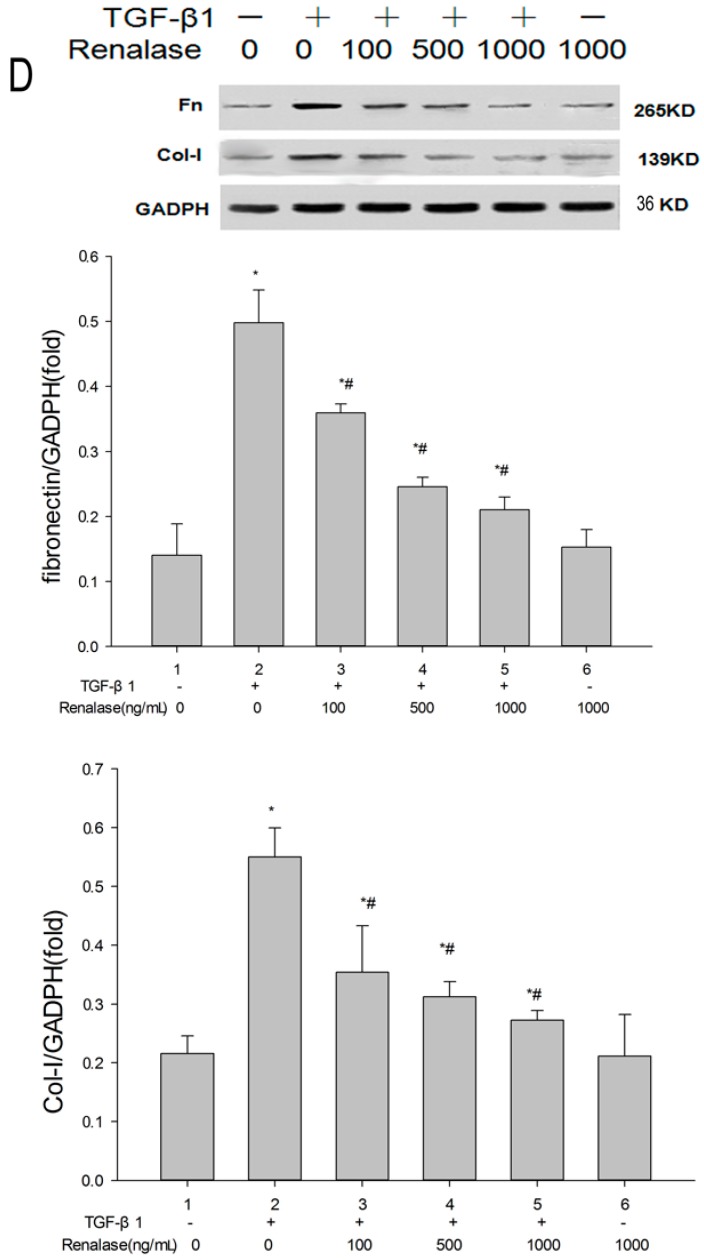
Renalase blocks transforming growth factor-β1 (TGF-β1)-mediated epithelial to mesenchymal transition (EMT) in vitro. Human proximal tubular epithelial cells (HK-2) were treated with 2 ng/mL TGF-β1 in the presence or absence of various concentrations of renalase as indicated for 48 h. (**A**) Immunofluorescence showed that renalase (1000 ng/mL) abolished TGF-β1-induced α-smooth muscle actin (α-SMA) assembly and preserved E-cadherin integrity; magnification: 120×; (**B**,**D**) Western blotting demonstrated that renalase (100, 500 and 1000 ng/mL) reversed the increased expression of α-SMA, collagen-I, and fibronectin, and decreased expression of E-cadherin in a dose-dependent manner; (**C**) reverse transcription (RT)-PCR revealed that renalase (100, 500 and 1000 ng/mL) reversed the increased Messenger RibonucleicAcid (mRNA) expression of α-SMA and decreased mRNA expression of E-cadherin in a dose-dependent manner. Results are presented as percentages of control values after normalization to GADPH and are the means ± SD of three independent experiments. * *p* < 0.05, compared with control groups; ^#^ *p* < 0.05, compared with TGF-β1-stimulated groups; *n* = 3 per group.

To obtain direct evidence that renalase can target renal fibrosis, we used an in vitro cell culture system in which human proximal tubule epithelial cells (HK-2) were induced to undergo EMT, an important mechanism of renal fibrosis, by TGF-β1. After treatment with TGF-β1, immunofluorescence showed that HK-2 cells began to lose the epithelial adhesion receptor E-cadherin and gained the mesenchymal marker α-SMA. However, simultaneous treatment with renalase abolished TGF-β1-induced α-SMA expression and assembly, and restored E-cadherin expression (Figure 3A). Similarly, Western blot and reverse transcription (RT)-PCR analysis showed that the decreased expression of E-cadherin and increased expression of α-SMA induced by TGF-β1 in HK-2 cells were significantly reversed by renalase at the protein and Messenger RibonucleicAcid (mRNA) level in a dose-dependent manner (Figure 3B,C). Furthermore, we investigated FN and Col-I expression to evaluate the degree of fibrosis. Consistent with the EMT results, renalase attenuated TGF-β1-induced FN and Col-I expression in a dose-dependent manner (Figure 3D).

### 2.4. Renalase Inhibits TGF-β1 Mediated EMT by a Non-Canonical Mechanism

**Figure 4:**
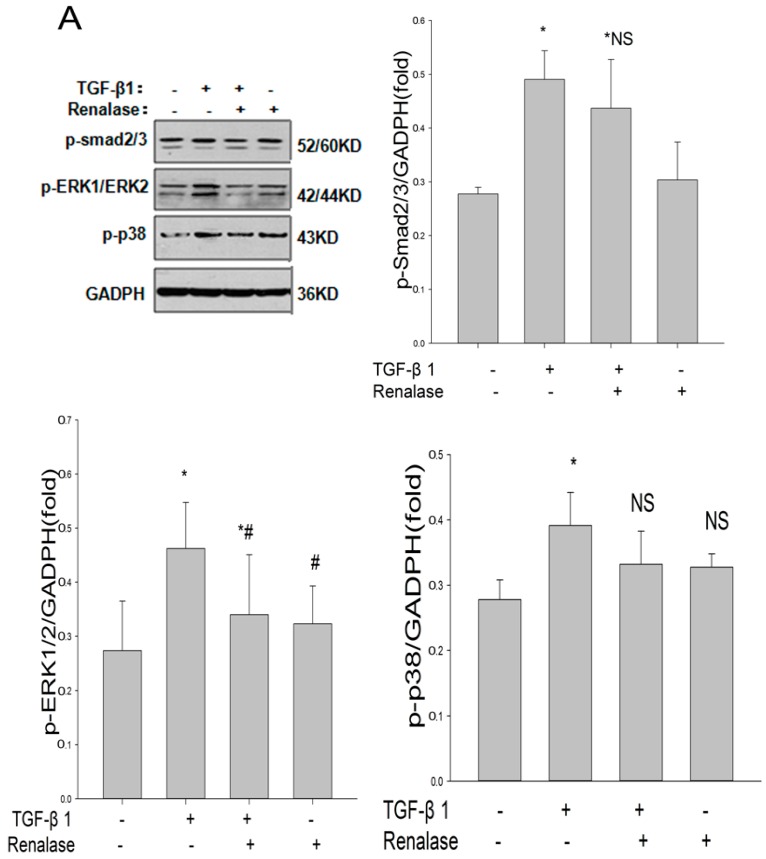

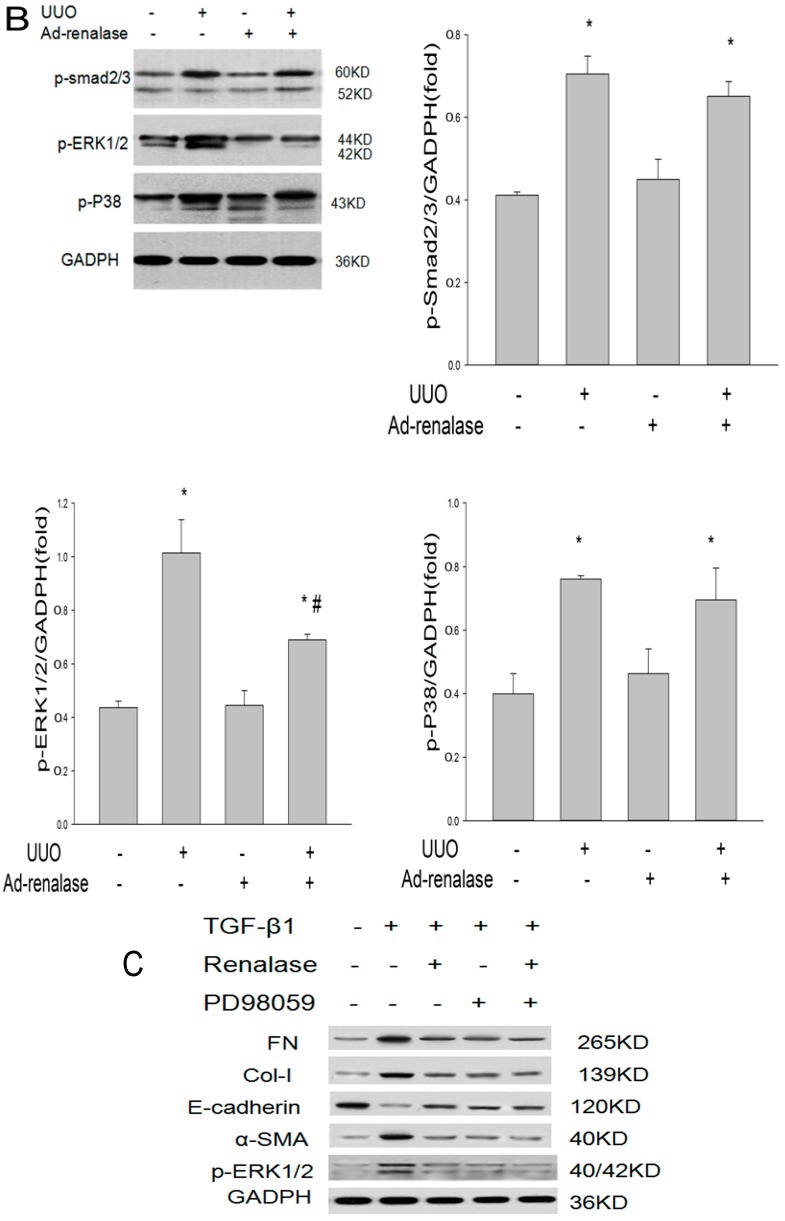

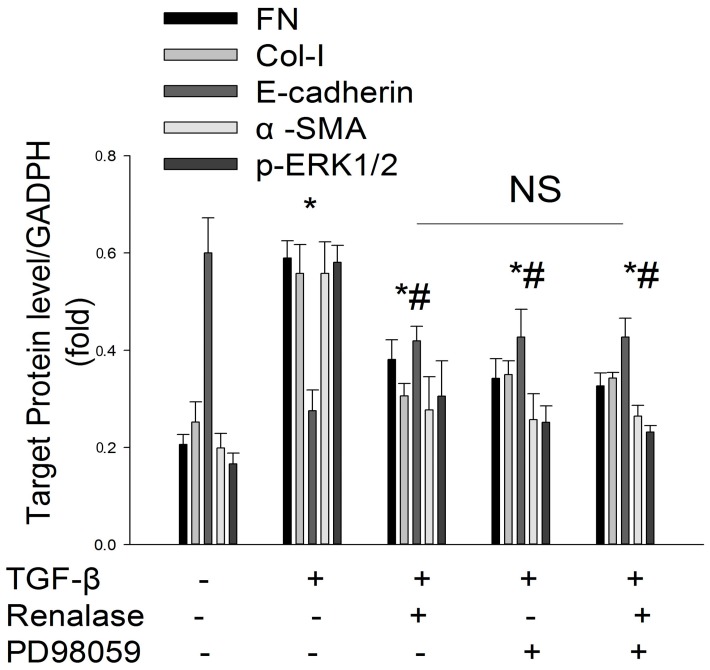
Renalase inhibits the TGF-β1-mediated increase of phosphorylated ERK1/2 in vitro. (**A**) Human proximal tubular epithelial cells (HK-2) were treated with 2 ng/ml TGF-β1 in the presence or absence of renalase (1000 ng/mL) for 48 h. Western blotting revealed that renalase inhibited TGF-β1-induced ERK1/2 phosphorylation, and did not affect the phosphorylation of Smad2/3 and p38. Results are presented as percentages of control values after normalization to GADPH and are the means ± SD of three independent experiments. * *p* < 0.05, compared with control groups; ^#^ *p* < 0.05, compared with TGF-β1-stimulated groups; NS: not statistically significant compared with control groups. *n* = 5 per group; (**B**) Changes of TGF-β1 signaling pathway in UUO rats. Renalase inhibited ERK1/2 phosphorylation and did not affect the phosphorylation of Smad2/3 and p38. Results are presented as percentages of control values after normalization to GADPH and are the means ± SD of three independent experiments. * *p* < 0.05, compared with sham + Ad-b-gal groups; ^#^ *p* < 0.05, compared with UUO + Ad-renalase groups; *n* = 5 per group; (**C**) Human proximal tubular epithelial cells (HK-2) were treated with 2 ng/mL TGF-β1 in the presence or absence of renalase (1000 ng/mL) or PD98059 (20 μM) for 48 h. Western blotting showed that renalase has a similar effect with PD98059 to minimize the increase of FN, Col-I, α-SMA and ERK1/2 phosphorylation and restore the decrease of e-cadherin induced by TGF-β1. While renalase and PD98059 were given at the same time to TGF-β1-stimulated cells, the protective effect of renalase and PD98059 was not superimposed. Results are presented as percentages of control values after normalization to GADPH and are the means ± SD of three independent experiments. * *p* < 0.05, compared with control groups; ^#^ *p* < 0.05, compared with TGF-β1-stimulated groups; NS: not statistically significant between groups. *n* = 5 per group.

To better understand the molecular mechanism of the inhibitory effects of renalase, we investigated the possible involvement of key TGF-β1 signaling pathway molecules in EMT. The results demonstrated that compared with the TGF-β1 alone group, there was no significant change in phosphorylated Smad2/3 and phosphorylated p38 expression when incubated with renalase, however the level of phosphorylated extracellular regulated protein kinases 1/2 (ERK1/2) was markedly decreased (Figure 4A). Then, we compared the effects of renalase with PD98059 on EMT and fibrosis. PD98059 is a selective inhibitor of MEK1 (a mitogen-activated protein kinase (MAPK)) which inhibits Erk1/2 phosphorylation and activation. The results showed that both renalase and PD98059 can partially attenuate EMT and fibrosis induced by TGF-β1 in vitro, however, there is no difference between the effectiveness of renalase and PD98059 (Figure 4C). In addition, while renalase and PD98059 were given at the same time to TGF-β1-stimulated cells, the protective effect of renalase and PD98059 was not superimposed (Figure 4C). This showed that the mechanism of renalase and PD98059 to protect fibrosis may be the same, that is, by inhibiting the role of ERK phosphorylation. Thus, renalase inhibition of TGF-β1-mediated EMT and fibrosis may be related to inhibition of activation of the ERK1/2 pathway. Moreover, we also detected the expression of TGF-β1 signaling molecules in rats. Consistent with the in vitro results, we found that the activation of the ERK signaling pathway was significantly inhibited by Ad-renalase in vivo (Figure 4B).

### 2.5. Role of ERK Signaling Pathway in the Inhibition of EMT by Renalase

**Figure 5:**
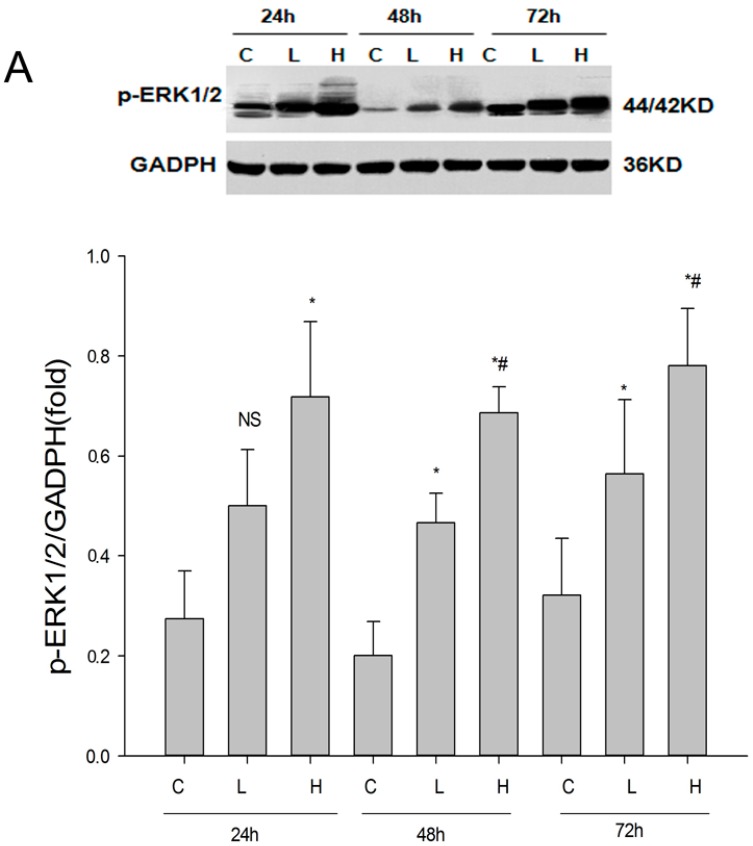

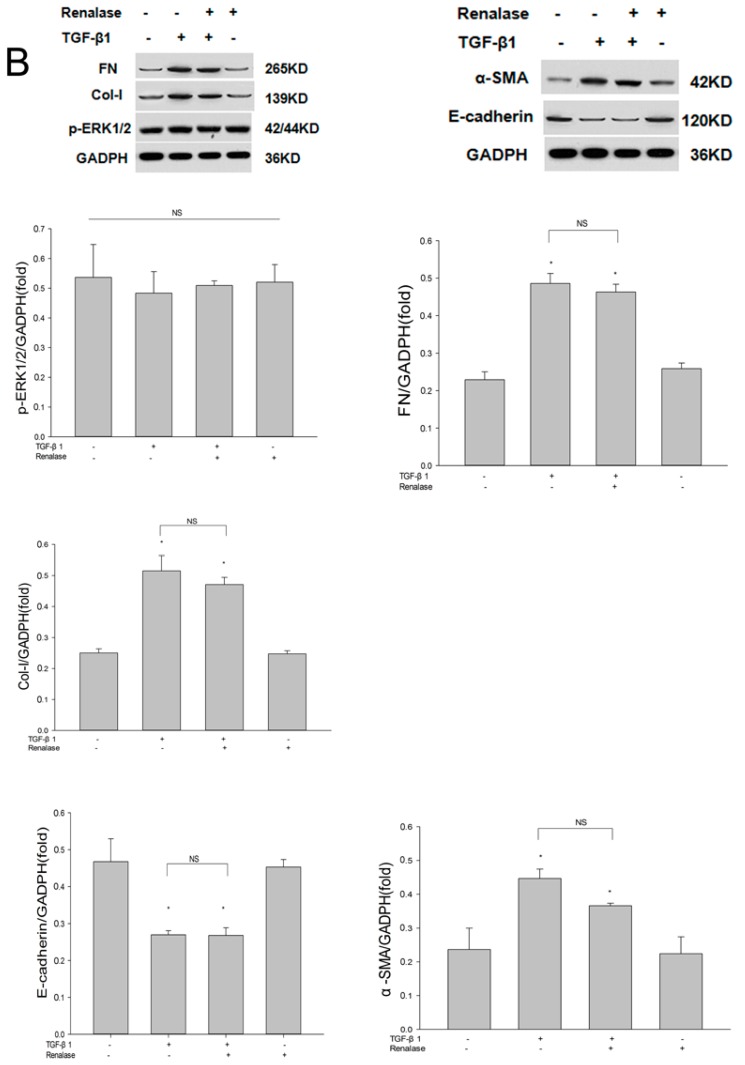

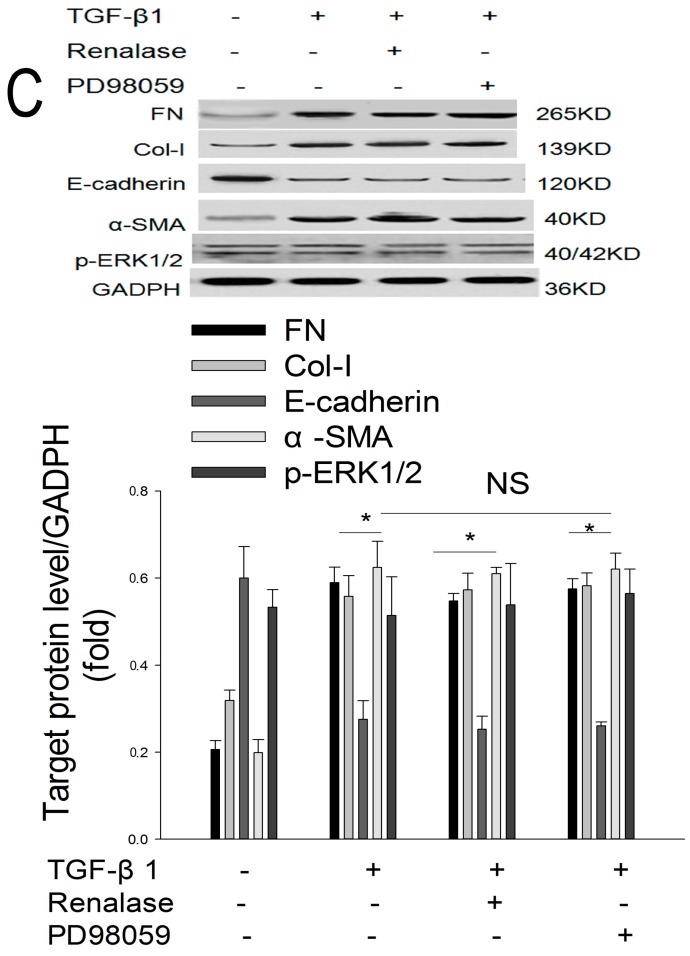
The role of ERK signaling pathway in the inhibition of EMT by renalase. (**A**) Results of plasmid transfection to overexpress ERK1. The transfection efficiency was observed with different doses of transfection reagents: low dose and high dose. Compared with control groups, the expression of p-ERK1/2 in the plasmid transfection groups were significantly higher than controls and the expression in the high dose group was higher than in the low dose group at 48 and 72 h. * *p* < 0.05, compared with control groups; ^#^ *p* < 0.05, compared with low dose groups. C: control group; L: low dose group; H: high dose group; NS: not statistically significant; (**B**) Effect of renalase on EMT and fibrosis induced by TGF-β1 in transfected cells. HK-2 were treated with plasmid and high dose transfection reagents in the presence or absence of TGF-β1 (2 ng/mL) and renalase (1000 ng/mL) as indicated for 48 h. Western blotting demonstrated that renalase cannot inhibit the increased expression of α-smooth muscle actin, collagen-I, and fibronectin and reverse the decreased expression of E-cadherin induced by TGF-β1 when ERK is overexpressed. * *p* < 0.05, compared with control groups; NS: not statistically significant between groups; (**C**) Effect of renalase and PD98059 on EMT and fibrosis induced by TGF-β1 in transfected cells. HK-2 were treated with plasmid and high dose transfection reagents in the presence or absence of TGF-β1 (2 ng/mL) and renalase (1000 ng/mL) or PD98059(20 µM) as indicated for 48 h. Western blotting demonstrated that the expression levels of p-ERK1/2 between groups were similar and that neither renalase nor PD98059 can inhibit the increased expression of α-smooth muscle actin, collagen-I, fibronectin or reverse the decreased expression of E-cadherin induced by TGF-β1 when ERK is overexpressed. * *p* < 0.05, compared with control groups. Results are presented as percentages of control values after normalization to GADPH and are the means ± SD of three independent experiments; *n* = 3 per group.

To further clarify the role of the ERK1/2 signaling pathway, we used plasmid transfection to overexpress ERK in HK-2 cells. The transfection efficiency was observed with different doses of transfection reagents, low dose and high dose. After transfection for 24 h, compared with other groups the levels of p-ERK1/2 in the plasmid transfection groups were significantly higher by western blot. However, the difference in expression of p-ERK1/2 between the low group and control group was not statistically significant (*p* = 0.063). With the extension of transfection time, compared with the control group the protein expression of the low transfected group was greatly increased, and the high dose group remained significantly higher than the low dose group (Figure 5A). We then stimulated the transfected cells with TGF-β1 to observe whether renalase could still inhibit EMT induced by TGF-β1. Results showed that the ability of renalase to inhibit TGF-β1-induced EMT and fibrosis was offset when transfected cells overexpressed ERK1 (Figure 5B). In addition, neither renalase nor PD98059 could alleviate EMT and fibrosis induced by TGF-β1 (Figure 5C). This indicated that renalase acts as an inhibitor of ERK1/2 phosphorylation to reduce EMT and fibrosis induced by TGF-β1.

## 3. Discussion

Progressive interstitial fibrosis is the main pathological feature of almost all forms of CKD [18]. Because of the limited efficacy of current therapies, it is necessary to explore new agents that can prevent the occurrence of renal fibrosis to prevent the disease from progressing. Renalase is a recently described endogenous signaling molecule which protects against cardiac and renal dysfunction. Furthermore, researchers found renalase reduces renal and cardiac fibrosis in the subtotal (5/6) nephrectomy model of cardiorenal syndrome [19]. Thus, it is conceivable that renalase may also play a role in preventing other forms of renal fibrosis. In the present study, we demonstrated that after UUO, a model with predominant tubulointerstitial lesions, renalase expression in the injured kidney underwent marked downregulation. Supplementation of renalase expression using an adenoviral vector suppressed the expression of interstitial matrix components and ameliorated renal fibrogenesis. An in vitro experiment was then carried out to explore the possible mechanism. Renalase can inhibit TGF-β1-induced EMT in proximal tubule epithelial cells, which is an important event in the occurrence of renal interstitial fibrosis. This effect was mediated by interference with the non-canonical TGF-β1 pathway, via inhibition of activation of the ERK1/2 pathway, an important regulatory signaling pathway in tubular EMT and renal fibrotic processes. These results suggest that the novel protein renalase may have potential therapeutic value in the treatment of fibrotic kidney disease and slowing of CKD progression.

Renalase, a secreted flavoprotein, not only protects against hypertension but also exerts cardio-renal-protective effects. Mounting evidence suggests that renalase exerts its cytoprotective effects by interacting with its plasma membrane receptor, not metabolizing catecholamines [7,20]. It is thought that renalase is secreted by renal tubular epithelial cells and the level of renalase in the circulation is closely related to the size of the kidney and renal function [21]. Although renal function was normal in UUO, the renal cortex was thinned and renal tubular epithelial cells underwent atrophy, transdifferentiation and loss of function, while fibrosis increased. This may be the reason why renalase expression was reduced. To investigate whether the decrease of renalase is related to the occurrence of fibrosis, we used an adenovirus to overexpress renalase and increase the level of renalase in kidney. The results demonstrated that renalase prevented the transformation of epithelial cells and overproduction of interstitial matrix components in the renal parenchyma. This is consistent with the results of previous studies. First, it was found that renalase can regulate blood pressure [9]. Hypertensive kidney disease is characterized by glomerular and interstitial fibrosis, tubular atrophy, and inflammation [22] and the degree of interstitial fibrosis strongly correlates with the rapidity of the progression of chronic kidney disease [23]. Hypertension and renal fibrosis are the cause and effect of each other. Therefore, renalase may reduce fibrosis through reduced blood pressure. However, we found that although blood pressure was slightly decreased when renalase was overexpressed, the difference was not statistically significant. The reason may be that unilateral ureteral ligation does not cause high blood pressure, and for the normal blood pressure rats renalase has no significant antihypertensive effect. Second, it is known that oxidative stress and the inflammatory response are important drivers for the development of renal interstitial fibrosis [24,25]. It was confirmed that renalase can reduce oxidative stress and inflammation in other animal models [14,17], which may be the underlying mechanism by which renalase reduces fibrosis. Whether oxidative stress and inflammation play a role in UUO needs to be confirmed. Third, in a recent study, researchers found renalase protected against renal injury and cardiac remodeling after subtotal (5/6) nephrectomy [19], which is a classical model of CKD, and the residual kidney would undergo significant fibrosis. It was confirmed that renalase could attenuate renal fibrosis, consistent with our results, which suggests that renalase may be of great significance in delaying the progression of CKD. However, not all research results are consistent. It was also found that renalase is a novel target gene of hypoxia-inducible factor-1α (HIF-1α) [15,26]. However, endothelial HIF-1α is essential in initiating glomerular injury and progression to renal fibrosis by the transcriptional activation of genes encoding multiple vasoactive proteins [27]. Moreover, HIF-1α may promote EMT by regulating fibrotic gene expression during ischemia/reperfusion injury in HK cells [28]. These results suggested that renalase may be positively associated with renal injury as a downstream gene of HIF-1α. This is not consistent with our results, and is probably because of the different models used. That is, HIF-1α initiated the transcription of the downstream gene in hypoxic conditions, but not in normoxic conditions. The problem remains to be further studied.

Whether renal fibrosis occurrs or not depends on fibroblast survival and the balance between extracellular matrix production and degradation. There is growing evidence that renal tubular epithelial cells as a major component of renal parenchyma are not simply bystanders, which played a decisive role in the development of renal fibrosis [29]. Upon stimulation by profibrotic cues such as TGF-β1, renal tubular epithelial cells undergo phenotypic transformation to interstitial fibroblasts and myofibroblasts through EMT. Therefore, inhibition of renal tubular epithelial cell EMT is an important goal to prevent renal fibrosis. Successful suppression of EMT means the suppression of mesenchymal markers and preservation of important epithelial proteins, which maintain the normal morphology and functions of epithelial cells [30,31]. In this context, the ability of renalase to attenuate the expression of α-SMA and restore the expression of E-cadherin in HK-2 cells, as shown in our study, underscores that renalase effectively protects against tubular EMT. Moreover, we have demonstrated that treatment with renalase directly blocks the ERK pathway, but not the TGF-β1/Smad pathway, in tubular EMT and renal fibrosis. Renalase interacts with a plasma membrane receptor(s) to mediate protein kinase B and the mitogen-activated protein kinase (MAPK) pathway. These signaling properties are critical to its cytoprotective effects. However, questions remain. In the setting of AKI, renalase may play a protective role through the activation of MAPK signaling pathway [7], although we found that renalase may alleviate fibrosis by inhibiting the activation of MAPK signaling pathway, consistent with Yin et al. [19]. The reason for the inconsistency may be differences in the disease model, stimulus and stimulation time. In addition, compared with the classic TGF-β1/Smad pathway, the activation of MAPK signaling pathway is not the main mechanism in TGF-β1-induced fibrosis. However, we observed that renalase significantly reduced EMT and fibrosis. Whether there are other mechanisms involved remains to be further explored. Importantly, although renal fibrosis is a common pathway for the progression of CKD to ESRD, the mechanism inducing renal interstitial fibrosis is not always the same due to different underlying causes of CKD. Thus, while the UUO model is a classic model of fibrosis, it cannot represent all types of fibrosis present in CKD. Therefore, just because renalase can reduce fibrosis caused by unilateral ureteral ligation does not necessarily mean it will be effective for all forms of fibrosis. Hence, further studies need to be carried out in different renal disease models. Moreover, further research is needed to determine whether there are any other mechanisms, such as the prevention of oxidative stress, inflammation or apoptosis, involved in the antifibrotic effect of renalase. 

## 4. Materials and Methods

### 4.1. Animal Models

Eight-week-old male Sprague-Dawley rats (weighing approximately 160–180 g) were obtained from the Institute of Laboratory Animal Science (Chinese Academy of Medical Sciences, Beijing, China). All rats were housed in a 12-h light/dark cycle with free access to water and fed with standard rat chow. Animals were randomly assigned to four groups (*n* = 5 per group): (1) sham + vehicle; (2) UUO + vehicle; (3) UUO + renalase; or (4) sham + renalase. While undergoing surgery, the rats were given the following treatments: (1) vehicle-operated treated with tail vein injection of 1.0 × 10^10^ Plaque Forming Units (PFU) control adenovirus (sham + Ad-b-gal or UUO + Ad-b-gal); or (2) renalase treated with tail vein injection of 1.0 × 10^10^ PFU adenovirus-renalase (sham + Ad-renalase or UUO + Ad-renalase). The effect of renalase gene delivery on the kidney was assessed 3, 7, and 14 days after adenovirus injection. Based on the test results, if necessary, a second dose was given. UUO was carried out using an established protocol [32]. Briefly, the rats were anaesthetized with sodium pentobarbital (50 mg/kg body weight) and the left ureter was double ligated with suture thread. Sham-operated rats had their ureters exposed, but not ligated. After surgery, the rats’ blood pressure was examined daily. All the animals were killed at specific times after UUO, that is 7, 10, 14, or 21 days to choose the most obvious time point for EMT and fibrosis and 14 days to conduct relative experiments. All of their kidneys and blood harvested for various analyses when the animals were killed. The animal study protocol conformed to the Guide for the Care and Use of Laboratory Animals published by the US National Institutes of Health and was approved by the Animal Experimentation Ethics Committee of Beijing Friendship Hospital (16-2001, 29 March 2016).

### 4.2. Cell Culture and Treatments

Human proximal renal tubular epithelial (HK-2) cells were obtained from the American Type Culture Collection (Manassas, VA, USA). The cells were cultured in Dulbecco’s modified Eagle medium (Sigma-Aldrich, St. Louis, MO, USA) supplemented with 5% fetal bovine serum (Gibco, Grand Island, NY, USA) as described previously. After serum starvation for 12 h, HK-2 cells were incubated with various concentrations of renalase (100, 500, or 1000 ng/mL; Cloud-Clone Corp., Houston, TX, USA) in the absence or presence of TGF-β1 (2 ng/mL; R&D Systems, Minneapolis, MN, USA) or PD98059 (20 µM; Sigma) for 48 h unless indicated otherwise. The cells were collected for various analyses. 

### 4.3. Transient Transfection

HK-2 cells at 50% confluency were placed in fresh culture medium containing 5% fetal bovine serum 2 h before transfection. Then, a plasmid containing human ERK1/MAPK3 (RC204196, Origene, Rockville, MD, USA) was transfected into the cells with Lipofectamine^®^ 3000 Transfection Reagent (Invitrogen, Burlington, ON, Canada) following the manufacturer’s protocol. In each experiment, efficiency of gene overexpression was measured by western blot analysis.

### 4.4. Immunofluorescence

Immunofluorescence was executed as described previously [33]. Briefly, cells cultured on cover slips were fixed with 4% paraformaldehyde and incubated with primary antibodies against α-smooth muscle actin (α-SMA) (ab5694; Abcam, Cambridge, MA, USA) or E-cadherin (sc-7870; Santa Cruz Biotechnology, Dallas, TX, USA). Frozen sections of kidney tissues were fixed with cold acetone, and then incubated with primary antibodies against α-SMA or E-cadherin. Then, the cells and cryosections were stained with secondary antibodies conjugated to Alexa Fluor 488 and mounted. Images of the cells and cryosections were obtained by confocal laser scanning microscopy (TCS SP5; Leica, Mannheim, Germany). The fluorescence intensity was detected by confocal laser scanning microscopy immediately.

### 4.5. Semi-Quantitative Assessment of Renal Fibrosis

To evaluate the extent of fibrosis, sections of paraffin-embedded kidney tissue were subjected to Masson’s trichrome staining (MTS) using a standard protocol [32]. Stained sections were examined under an Eclipse E600 epifluorescence microscope equipped with a digital camera (Nikon, Melville, NY, USA). The severity of renal fibrotic lesions was defined as the percentage of MTS-positive areas and analyzed by the Image Acquisition and Analysis Software LabWorks (Ultra-Violet Products, Cambridge, UK). For each sample, we analyzed five randomly selected nonoverlapping fields.

### 4.6. Immunohistochemistry

Paraffin-embedded sections of kidney tissue were incubated with primary antibodies against fibronectin (FN) (ab2413; Abcam), collagen I (Col-I) (ab34710; Abcam), or renalase (ab178700; Abcam) overnight at 4 °C. Detection of antibody binding was performed using an ABC ELITE kit (Vector Laboratories, Burlingame, CA, USA) including biotinylated secondary antibodies according to the manufacturer’s instructions. Sections that were stained with secondary antibodies alone were confirmed to be negative. Image acquisition was performed using the analysis system described above. Quantification of immunohistochemistry was performed using Image-Pro Plus software (Media Cybernetics, Rockville, MD, USA). Optical density (IOD) = density (mean) × area; density is the concentration or intensity of the reaction-positive protein. Mean density (MOD) = IOD/(SUM× area). 

### 4.7. Quantitative Real-Time RT-PCR

Total RNA was isolated with Trizol reagent (Invitrogen) and methyl trichloride according to the manufacturer’s instructions. RNA concentration and quality were checked by spectrophotometry. First-strand cDNA was synthesized from 2 μg RNA by reverse transcription using AMV-RT (Promega, Madison, WI, USA) and random primers at 42 °C for 30 min. Quantitative reverse transcription polymerase chain reaction (RT-PCR) was performed on an ABI PRISM 7000 sequence detection system (Applied Biosystems, Foster City, CA, USA). The 25-μL reaction mixture included 12.5 μL 2× SYBR Green PCR Master Mix (Applied Biosystems), 5 μL diluted cDNA (1:10), and 0.5 μM sense and antisense primers. The primers were designed using Primer Express software v.2.0 (Applied Biosystems) and their sequences are provided in Supplementary Table S1. Amplification was carried out under the following conditions: initial denaturation for 10 min at 95 °C, denaturation for 10 s at 95 °C, annealing for 30 s at an optimal temperature for each primer pair, and extension for 30 s at 72 °C. The mRNA levels of target genes were calculated after normalization to β-actin mRNA.

### 4.8. Western Blot Analysis

Preparation of whole cell lysates and kidney tissue homogenates, and immunoblotting were performed as previously described [32]. The primary antibodies were obtained as follows: anti-α-SMA (ab5694; Abcam), anti-FN (ab2413; Abcam), anti-col-I (ab138492; Abcam), and anti-E-cadherin (sc-7870; Santa Cruz Biotechnology or ab15148; Abcam). Anti-p-Smad2 (Ser465/467)/Smad3 (Ser423/425) (8828), anti-p-Erk1/2 (Thr202/Tyr204) (9101), and anti-p-p38MAPK (Thr180/Tyr182) (4511) were obtained from Cell Signaling Technology (Danvers, MA, USA).

### 4.9. Enzyme-Linked Immunosorbent Assay (ELISA)

The plasma levels of renalase in rats were evaluated by ELISA. Venous blood samples were collected in Ethylenediaminetetraacetic acid (EDTA)-/acetic acid-containing tubes and centrifuged at 3000 rpm for 10 min. The plasma was collected (and frozen at −80 °C until analysis. Renalase assays were performed using a renalase-96 microplate ELISA kit (SEC845Ra, Cloud-Clone Corp.) according to the manufacturer’s instructions.

### 4.10. Statistical Analyses

All data are expressed as the mean ± standard deviation (SD). Statistical analyses were performed using SPSS version 17.0 software (IBM-SPSS, Armonk, NY, USA). Comparisons between groups were made using one-way analysis of variance followed by the Student–Newman–Keuls test. *p* < 0.05 was considered to be statistically significant.

## 5. Conclusions

Collectively, our results suggest the antifibrotic effects of renalase are mediated through antagonism of the EKR1/2 signaling pathway, resulting in decreased extracellular matrix production and improved tubulointerstitial lesions. These data indicate that renalase behaves as a potential novel suppressor of renal fibrosis. Therefore, supplementation with exogenous renalase may be a promising strategy to slow or halt the development of CKD.

## Supplementary Materials

Supplementary materials can be found at www.mdpi.com/1422-0067/18/5/855/s1.

## Abbreviations

CKD	Chronic kidney disease
RIF	Renal interstitial fibrosis
ESRD	End-stage renal disease
EMT	Epithelial–mesenchymal transition
TGF-β1	Transforming growth factor-β1
AKI	Acute kidney injury
UUO	Unilateral ureteral obstruction
MTS	Masson’s trichrome staining
HK-2	Human proximal tubule epithelial cells
HIF-1α	Hypoxia-inducible factor-1α
MDPI	Multidisciplinary Digital Publishing Institute
DOAJ	Directory of open access journals
TLA	Three letter acronym
LD	Linear dichroism
