# Supplementary material for: Renalase Protects against Renal Fibrosis by Inhibiting the Activation of the ERK Signaling Pathways
Source: Int J Mol Sci. 2017 Apr 27;18(5):855. doi: 10.3390/ijms18050855 (PMC5454808; doi:10.3390/ijms18050855)
Supplement: Supplementary file 1 [file ijms-18-00855-s001.pdf]

Supplementary Materials: Renalase Protects against Renal Fibrosis by Inhibiting the Activation of the ERK Signaling Pathways

Yiru Wu, Liyan Wang, Dai Deng, Qidong Zhang and Wenhu Liu

Table S1. Sequences of the primers for Quantitative Real-Time (RT-PCR).

Primer	Sequence (5'to3')	Length (bp)
α-SMA_F	ACTGCCTTGGTGTGTGACAA	224
α-SMA _R	TCCCAGTTGGTGATGATGCC	
E-cadherin_F	TCATGAGTGTCCCCCGGTAT	240
E-cadherin _R	TCTTGAAGCGATTGCCCCAT	
β-actin_F	TGACGTGGACATCCGCAAAG	205
β-actin _R	CTGGAAGGTGGACDGCGAGG	

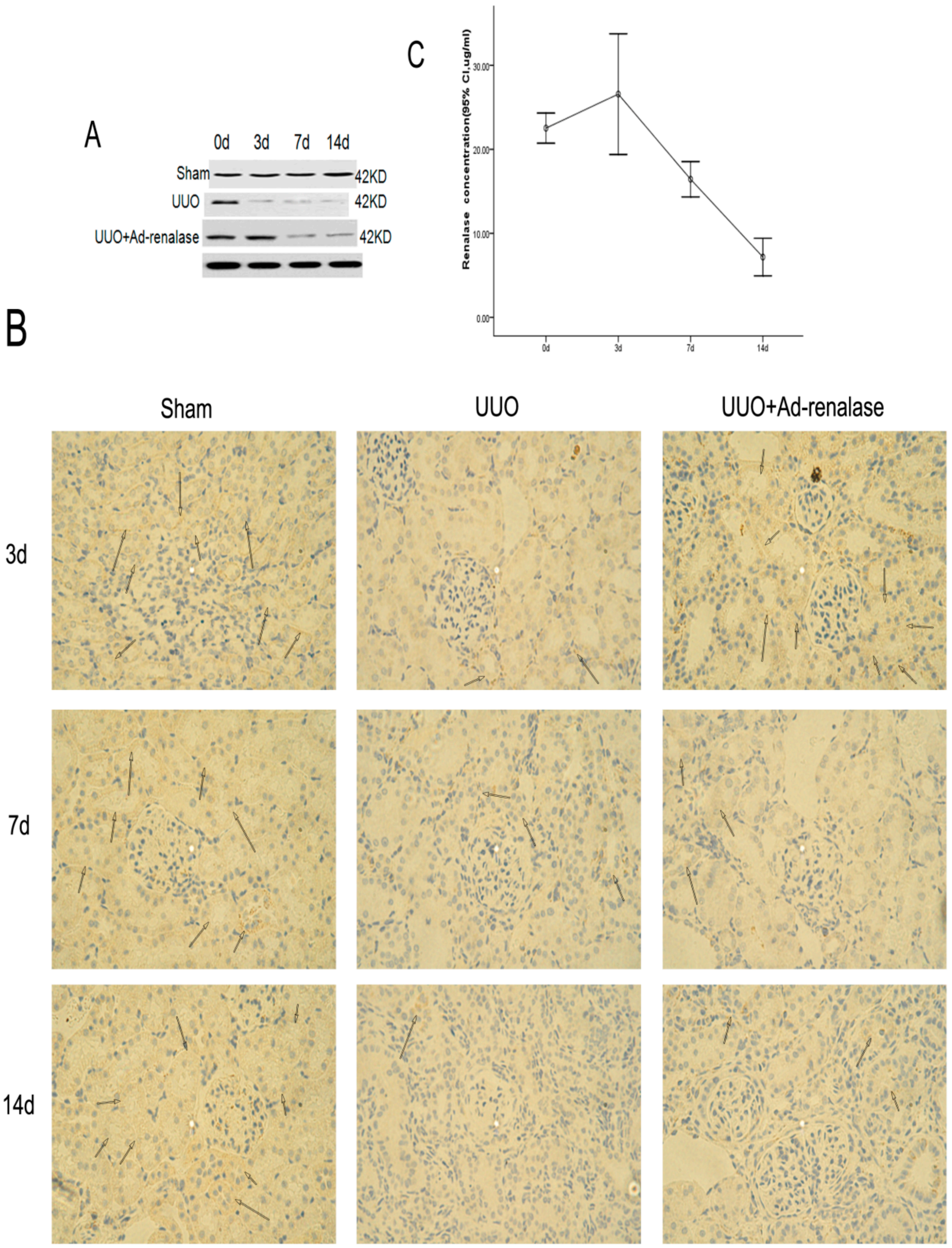

Figure S1. Renalase expression result after adenovirus injection. (**A**,**B**) Western blot and immunohistochemistry analysis showed the expression of renalase in different groups at different times. At 3 days after surgery, western blot and immunohistochemistry showed the expression of renalase was significantly decreased in the UUO group compared with the sham group, while in contrast, the expression of renalase was similar in the UUO + Ad-renalase group compared with the sham group. The expression of renalase in the UUO + Ad-renalase group declined at 7 days after surgery. Arrows refer to positive results in figure B. (**C**) Circulation concentration of renalase in the UUO + Ad-renalase group. After injection of renalase adenovirus, the expression of renalase in plasma was increased in 3 days after UUO, but then as time passed, the expression of renalase decreased. d: days.
